# Evaluations of a Detector-Limited Digital Impedance Bridge

**DOI:** 10.6028/jres.126.006

**Published:** 2021-04-06

**Authors:** Mona Feige, Stephan Schlamminger, Bryan Waltrip, Michael Berilla, Yicheng Wang

**Affiliations:** 1 National Institute of Standards and Technology, Gaithersburg, MD 20899, USA

**Keywords:** AC voltage ratio, digital bridge, impedance standard, lock-in detector, noise cancellation

## Abstract

We tested a simple digital impedance bridge using two nominally equal resistors to form a 1:1 ratio. We focused on resolution and stability of the detectors. Fluctuations of the source voltages were largely removed through postprocessing of the digitized data, and the measurement results were limited by the detector noise. This detector-limited operating condition was first demonstrated using three modified Keysight 3458A multimeters for measurements of the voltage ratios, achieving 0.01 μV/V type A uncertainty in less than 15 min at 1 kHz. In an effort to extend the applicable frequency range and develop a system with off-the-shelf components, we tested a system using three lock-in detectors for measuring small deviations from the perfect AC ratio of unity magnitude, achieving stabilities and resolutions of 0.1 μV/V in a few hours for each point from 1 kHz to 5 kHz.

## 1 Introduction

The evolution of AC measurement techniques for impedance comparisons has been recently reviewed [1]. While transformer-based impedance bridges still provide the measurements with the highest accuracy for the most demanding applications, including the realization of the capacitance unit from calculable capacitors or from the AC quantized Hall resistance through a quadrature bridge, the digital bridges [2–6] have been noticeably improving for impedance comparisons, offering many advantages through computer control and automation. Josephson arbitrary waveform synthesizers establish a quantum-based voltage ratio standard that can be used for impedance comparisons at any phase angle [1]. Digital signal sources custom-designed for impedance bridges have also shown great promise. A dual-channel AC voltage source with amplitude ratio stability better than 0.01 μV/V and phase resolution of 0.2 µrad at 1 kHz has been reported [4]. Particularly relevant to the present work, another interesting approach reported by Kürten Ihlenfeld and Vasconcellos [6] uses “run-of-the-mill” workbench synthesizers of poor accuracy and amplitude stability that are then stabilized with a negative feedback loop, minimizing the bridge error signal.

In a recent conference paper [7], we reported a digital sampling bridge using three Keysight 3458A^1^1 Certain commercial equipment, instruments, or materials are identified in this paper to foster understanding. Such identification does not imply recommendation or endorsement by the National Institute of Standards and Technology, nor does it imply that the materials or equipment identified are necessarily the best available for the purpose. multimeters for measurements of the voltage ratios. All three 3458As were modified to allow their reference oscillators to phase lock with an external 10 MHz reference, enabling the control software to calculate multimeter analog-to-digital converter (ADC) aperture times according to equivalent-time sampling principles [8, 9] to maximize both the effective number of bits and noise rejection. We showed that the digital bridge effectively suppresses the source noise by more than two orders of magnitude, and the residual noise is largely white detection noise that can be further reduced through averaging. We also achieved a stability and resolution of 0.01 μΩ/Ω in less than 15 min at 1 kHz for comparisons of two nominally equal resistors. The digital technique we employed complements the analog noise suppression technique described in Ref. [6]; however, it requires further studies to delineate its advantages and limitations. Our current software design for using 3458A as AC waveform digitizers works well only at a discrete set of frequencies constrained by the equivalent-time sampling principles; this limits its usefulness at important frequencies like 1592 Hz and 1233 Hz, which are often desirable for impedance comparisons.

**Fig. 1:**
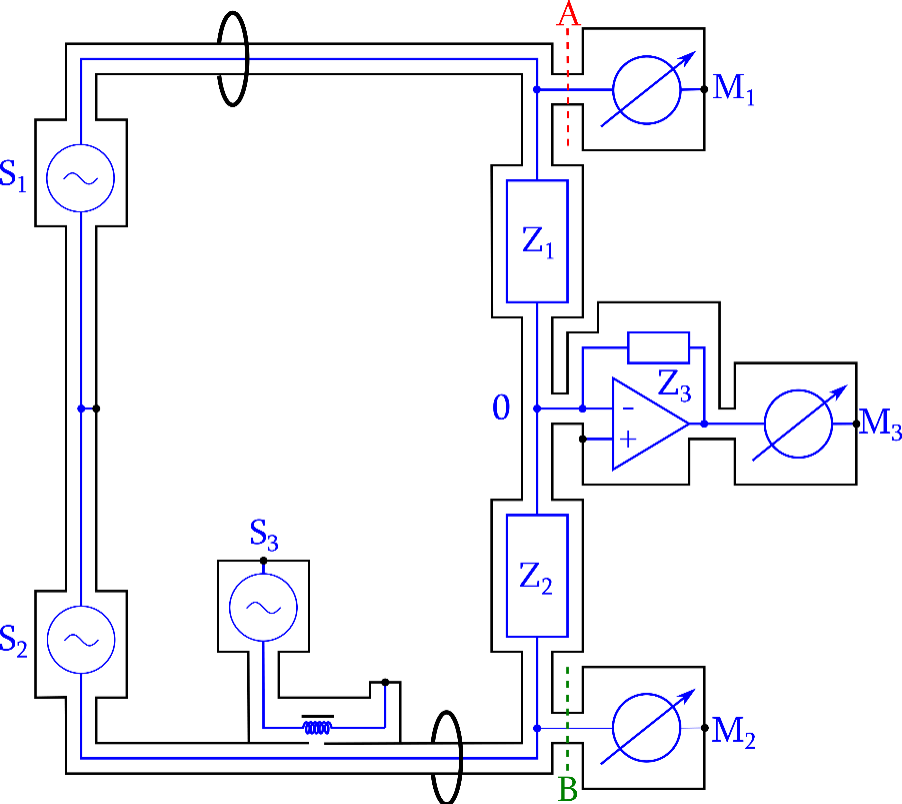
Schematic of digital impedance bridge. S_1_, S_2_ and S_3_: waveform generators. M_1_, M_2_ and M_3_: AC voltmeters. M_1_ and M_2_ connected to the high-potential ports (A and B) are periodically switched to minimize the effect of their gain drift. Z_1_ and Z_2_ are two resistors under comparison; Z_3_ is the feedback resistor of the current amplifier.

In this extended paper, we report the continued effort at the National Institute of Standards and Technology (NIST) to re-examine the digital approach, using a simple bridge setup as shown in Fig. 1. Here, we used Stanford Research Systems SR860 lock-in amplifiers to measure the AC voltage ratio. These commercial instruments have lower resolutions and stabilities compared to 3458A, but they are very robust and can be easily programmed and controlled remotely.

## 2 Bridge Setup

The digital bridge (Fig. 1) relies on accurate measurements of voltage ratios. In the ideal case, the excitation sources would be adjusted to balance the bridge, such that for any measured voltage, *V_2_*, at the high-potential port of *Z_2_*, the measured voltage, *V_1_*, at the high-potential port of *Z_1_* would be equal to a perfect value *V_1p_*, achieving the condition of equal current through the two impedances under comparison. The balance equation is

$\frac{Z_{1}}{Z_{2}}=-\frac{V_{1p}}{V_{2}}$ (1)

We used two phase-locked channels (S_1_ and S_2_) of a Keysight 33500B waveform generator as the main sources to excite the bridge. To overcome the limited resolutions of the generator outputs, another synchronized 33500B generator (S_3_) was used to inject a fine adjustment signal through a 100:1 injection transformer inserted into the lower excitation arm of the bridge. The residual imbalance voltage combined with the source drift can be represented by an error voltage, *δV*, superimposed on the ideal voltage *V_1p_*, and we have *V_1_=V_1p_+δV*. The error voltage is automatically balanced using a current amplifier (Femto DLPCA-200) with transimpedance of *Z_3_*. The common low-potential port, 0, is kept at virtual ground, and the detected error voltage, *V_3_*, relates to *δV* through:

$\frac{Z_{1}}{Z_{3}}=-\frac{\delta V}{V_{3}}$ (2)

The bridge dynamics can be understood as a superposition of the two voltage-balancing actions governed by Eq. (1) and Eq. (2).

**Fig. 2:**
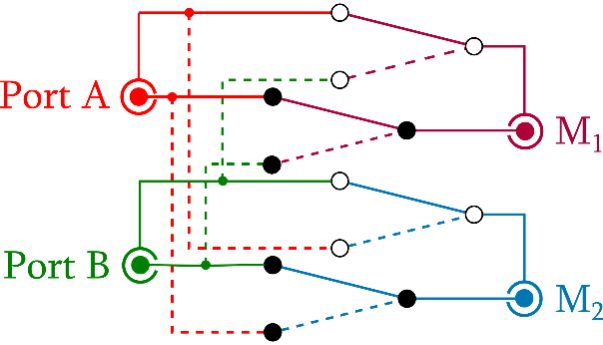
Schematic of coaxial switching box.

We used three SR860 lock-in detectors, M_1_, M_2_, and M_3_, in the float input mode for measurements of *V_1_*, *V_2_*, and *V_3_*, respectively. To achieve an overall bridge accuracy of 0.1 μΩ/Ω, the voltage ratio of *V_1_/V_2_* must be measured better than that value. The two main lock-in detectors connected to the high-potential ports (A and B) were periodically interchanged every minute, as illustrated in Fig. 2, using a custom coaxial switching fixture based on commercial coaxial relay modules (U74002-5PL, Universal Switching Corp.), to minimize the effect of their gain drift. A small loading change at A and B is equivalent to a small change of the excitation voltage ratio, which is suppressed in the digital domain by correlation with the bridge error signal. Then, *δV* only needs to be determined to better than 1 mV/V through measuring *V_3_*, since *δV/V_1_* is less than 10^−4^ in practice.

The bridge layout (Fig. 1) is influenced by the double-loop method promoted by Jeffery *et al.* [10] to use two-terminal-pair (2TP) bridges for four-terminal-pair (4TP) impedance comparisons, where a 2TP bridge connecting to the high-current ports excites the bridge, and another 2TP loop connecting through the high-potential ports forms a 2TP bridge for the voltage ratio measurements. In our case, the two detectors connecting to ports A and B, together with *Z_1_* and *Z_2_*, form a four-arm 2TP bridge for measuring the voltage ratio, enabling us to achieve the limiting condition where the overall bridge resolution and stability are limited by the detectors rather than the excitation sources, as is often the case in a typical 2TP digital bridge [11]. This feature also differentiates this bridge from the digital sampling bridges reported previously [12–14]. Those sampling bridges all had a single 2TP loop and employed a single detector to sample voltages at different potential ports; their performances largely depended on the source stabilities.

We used two Vishay resistors (HZ series) with a nominal value of 12.906 kΩ, closely matched within 0.3 μΩ/Ω, for *Z_1_* and *Z_2_* in our test bridge. The resistors’ low leads were soldered together, and the soldered point was connected to two British Post Office (BPO) connectors. One of the BPO connectors served as the common low-potential port to avoid the need of a combining network, while the other connector provided access to the low-current port of each 4TP resistor, which was needed for DC measurements.

For future comparisons between a resistor and a capacitor (RC), we may continue to omit the combining network by following a method used by Small *et al.* [15] to compare 4TP resistors with 2TP capacitors. The current amplifier was connected to the low-potential port of the 4TP resistor. Hence, the cable and the contact resistance between the low-current port of the resistor and the low port of the 2TP capacitor were then considered part of the capacitance standard. As long as the defining planes are applied consistently in calibrations, the inclusion of contact resistance only affects the dissipation factor of the capacitor slightly, with negligible contribution to the uncertainty of the capacitance measurements.

## 3 Test Results

### 3.1 Equal Voltage Test

**Fig. 3:**
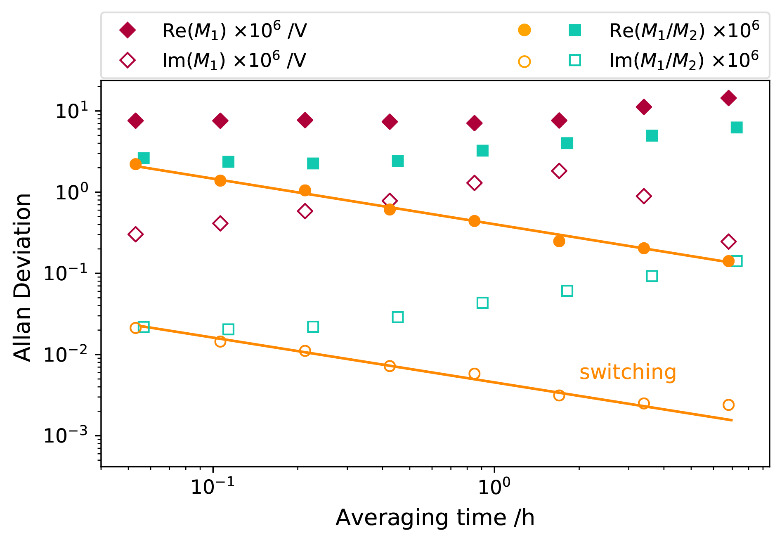
Allan deviations of a single source. Diamonds: measurements with one lock-in detector; squares: ratio of two detector measurements; circles: ratio measured with two virtually identical detectors created by periodical switching.

Lock-in detectors can be used to measure small AC signals down to the nanovolt range, but they are rarely used to measure large AC signals when low uncertainties are required. This is because the measurement accuracy is limited by the resolution of the ADC and the gain stability of the input amplifier. Typically, the front panel of the SR860 displays only four valid digits for the 1 V input range. To determine the limitations of using the lock-in detectors for voltage ratio measurements, we connected two SR860s in parallel to the same sinewave output of a 33500B at 1 kHz, with a root mean square (rms) value of 0.7071 V. The timing alignments of the two lock-ins were implemented in the control software using a M_1_-M_2_-M_2_-M_1_ sequence for the data readings. The Allan deviations of the source are shown in Fig. 3. The source instability was dominated by 1/*f* variations in amplitude, and the phase variations were much smaller (diamonds). The voltage ratio measurements showed smaller Allan deviations (squares), indicating that the detectors were more stable than the source, and the two SR860s could track the variations within 3 μV/V in a short period. The apparent increases of the Allan deviations after the initial decreases can be attributed to the slow gain drift of the lock-in detectors. The Allan deviations (circles) of the final test of a unity voltage ratio were acquired with two virtually identical detectors created by periodically switching the two lock-in detectors, yielding a straight line in the log-log plot. Its slope is consistent with averaging over white noise.

### 3.2 Digitized Bridge Voltages

**Fig. 4:**
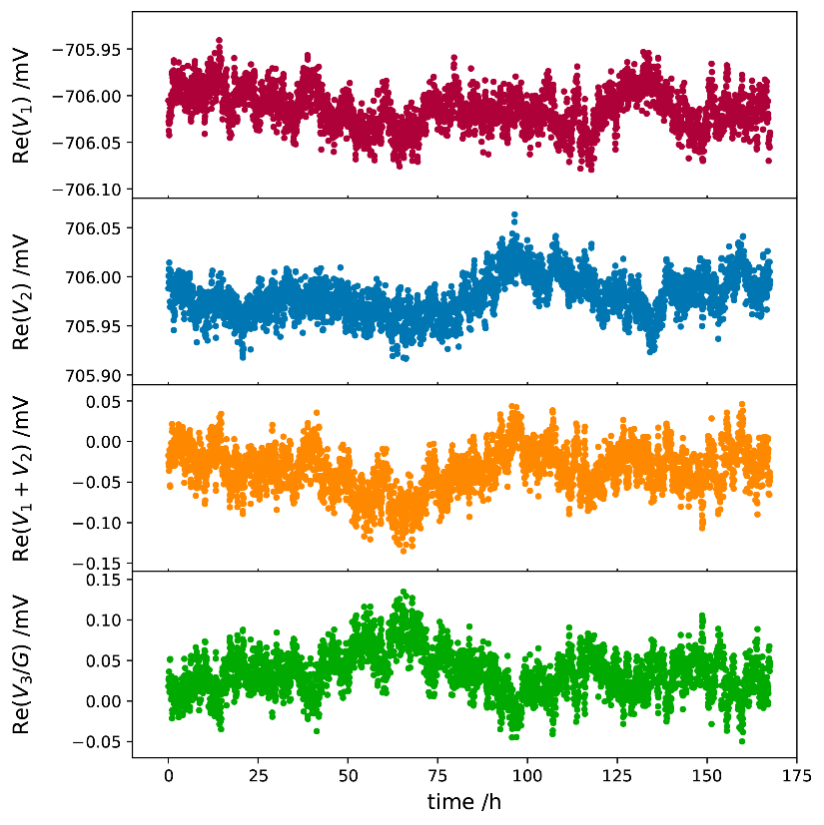
Real components of recorded voltages as function of time: (1) *V*_1_, (2) *V_2_*, (3) *V_1_+V_2_*, and (4) *V_3_* scaled with the gain (*G*) of the transimpedance amplifier.

**Fig. 5:**
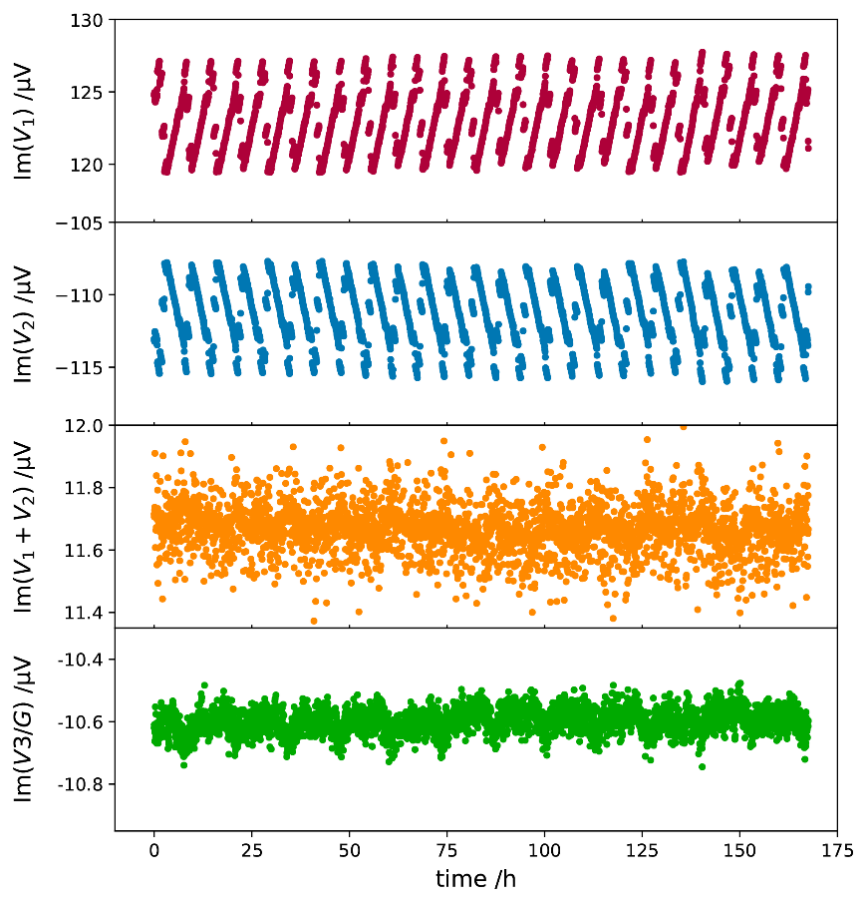
Imaginary components of recorded voltages as function of time: (1) *V*_1_, (2) *V_2_*, (3) *V_1_+V_2_*, and (4) *V_3_* scaled with the gain (*G*) of the transimpedance amplifier.

A major advantage of the digital bridge is that the excitation voltages and the error signal can be fully digitized, and the bridge dynamics can be analyzed in postprocessing. All the test results presented herein were acquired with the bridge setup shown in Fig. 1, with the two Vishay resistors in an air bath at 23 °C. The gain of the transimpedance amplifier was set at 10^7^ V/A, and the corresponding *Z_3_* was approximately 10 MΩ; the 3 dB bandwidth at this setting is 50 kHz. The timing alignments of the three lock-in detectors were implemented in the control software using an M_1_-M_2_-M_3_-M_2_-M_1_ sequence for the data readings. Figures 4 and 5 show the measured *V_1_*, *V_2_*, and *V_3_* values as a function of time that were acquired with S_1_ and S_2_ set at 1 kHz and an rms value of 0.7071 V. The reference phases of the three lock-in detectors were initially aligned using the excitation source driving *Z_2_*, and they were not disturbed in subsequent bridge balancing to minimize *V_3_*. The real parts of *V_1_* and *V_2_* (Fig. 4) fluctuated more than the imaginary counterparts (Fig. 5), reflecting the fact that the digital sources have better phase stabilities than amplitude stabilities. The phases of *V_1_* and *V_2_* showed a strong anticorrelation (Fig. 5). A strong amplitude correlation was also demonstrated by turning off the room-temperature control. In normal operation, the laboratory temperature fluctuates within 1 °C, and the amplitude correlation of *V_1_* and *V_2_* was not obvious, even when they were dominated by two output channels of the same waveform generator (Fig. 4).

The periodic phase fluctuations of *V_1_* and *V_2_* (Fig. 5) are interesting. Im(*V_1_*) and Im(*V_2_*) form mirror images of each other, and their periodic patterns disappear when they are summed together. The observed phase correlation remains even when *V_1_* and *V_2_* are driven by separate waveform generators. We speculate that the periodic phase fluctuations result from phase-locking actions inside M_1_ and M_2_, originating from the finite word length of the direct digital synthesizer that generates the reference signal.

We can qualitatively understand how the detected error voltage *V_3_* relates to the source fluctuation *δV* by considering that the transimpedance amplifier together with *Z_1_* and *Z_2_* form a summing amplifier. Since *Z_1_* and *Z_2_* are nominally equal, the input voltage to the summing amplifier is *V_1_+V_2_ ≈ δV*. As can be seen in Fig. 4, Re(*V_1_+V_2_*) closely matches with −Re(*V_3_*), where the latter is scaled with an estimated gain factor. Fluctuations of Im(*V_1_+V_2_*) and Im(*V_3_*) are smaller than their real counterparts by two orders of magnitude, and both are dominated by white noise.

### 3.3 Noise Cancellation and Results

To analyze the dynamics of the bridge balancing more rigorously, we applied Kirchhoff’s law to point 0 of the bridge circuit:

$\frac{V_{1}}{Z_{1}}+\frac{V_{2}}{Z_{2}}+\frac{V_{3}}{Z_{3}}=0$ (3)

Rearranging Eq. (3), we have:

$\frac{V_{1}}{V_{2}}+\frac{Z_{1}}{Z_{2}}+\frac{Z_{1}}{Z_{3}}\frac{V_{3}}{V_{2}}=0$ (4)

Using conventional notations, we define the impedance ratio:

$\frac{Z_{1}}{Z_{2}}=1+\alpha +i\beta$ (5)

Further, we define the gain factors between *Z_1_* and *Z_3_*:

$g=|g|e^{i\theta }\equiv \frac{Z_{1}}{Z_{3}}$ and G=1/g (6)

Equation (4) can then be rewritten as follows:

$\frac{V_{1}}{V_{2}}+1=-\alpha -i\beta -g\frac{V_{3}}{V_{2}}$ (7)

If the bridge system were noise free, and we had unlimited resolutions in *V_1_* and *V_2_*, then the voltages could be adjusted to achieve the balance condition ($V_{3}\;$= 0) and easily determine α and β. If the bridge system were noise free, but we have limited resolutions in *V_1_* and *V_2_*, then we need to operate the bridge at minimum of two different ratios of *V_1_/V_2_* so that the complex gain parameter g with a magnitude |g| and an argument θ can be determined first. In practice, the gain parameter is determined in two steps by analyzing the correlation between the fluctuating *V_1_/V_2_* and *V_3_/V_2_* values. Let us define:

$u=\frac{V_{1}}{V_{2}}+1$ (8)

and

$w=-|g|\frac{V_{3}}{V_{2}}$ (9)

**Fig. 6:**
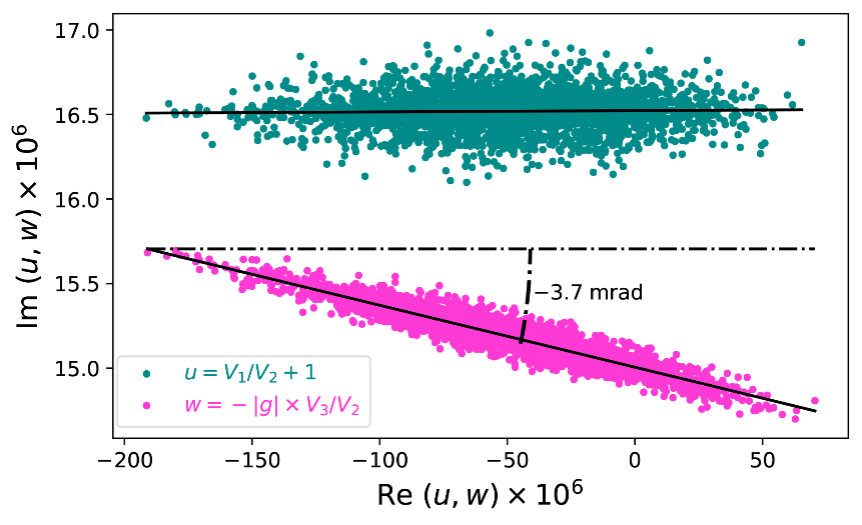
Imaginary part versus real part: *u* in green and *w* in purple. The obtained angle is independent of *|*g*|.*

To obtain the argument, we plot the imaginary part versus the real part for *u* and *w* at 1 kHz in Fig. 6. The fluctuation of *u* is mainly along the real axis, and the distribution of *w* exhibits a similar pattern except in a tilted angle. Line fitting to the two patterns allows us to determine the angle *θ*. Note the angle remains the same whether $\left|g\right|$ is known or not, because the real and imaginary parts of *V_3_/V_2_* are scaled by the same factor. In the figure, we use the approximate gain factor to demonstrate that u and w are about the same.

**Fig. 7:**
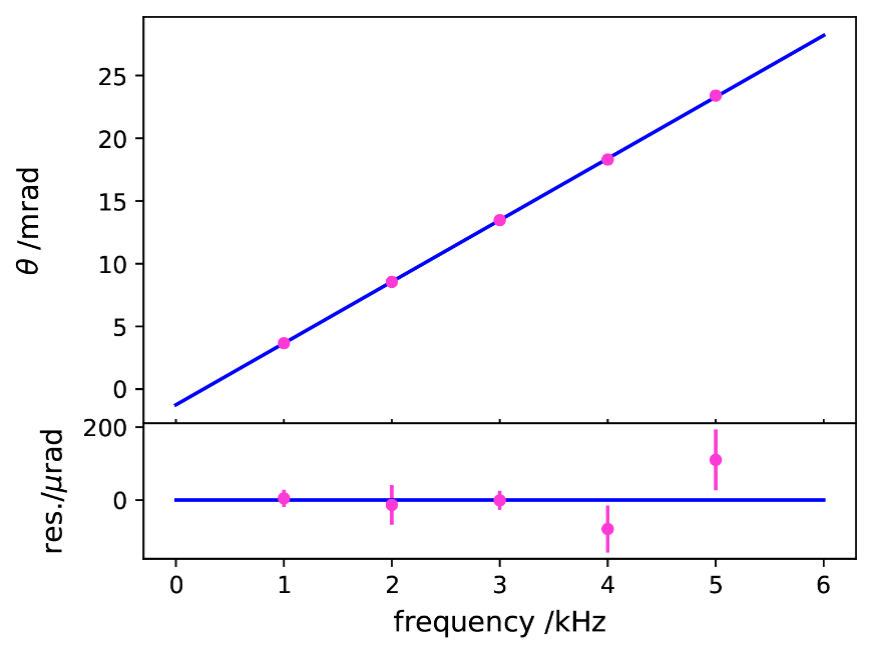
Phase shift of transimpedance amplifier as a function of frequency (top). Residual after fitting with a straight line (bottom).

Figure 7 shows the measured *θ* as a function of frequency from 1 kHz to 5 kHz. The linear frequency dependence can be understood by modeling *Z_3_* as a parallel RC network.

With the angle *θ* determined, it is helpful to further introduce:

$v={-e}^{i\theta }\frac{V_{3}}{V_{2}}$ (10)

**Fig. 8:**
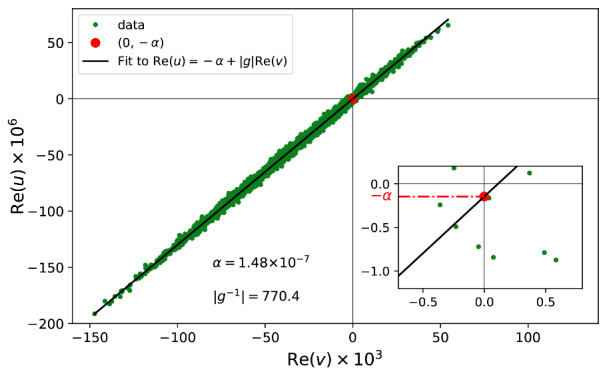
Least-square line fitting of Re*(u)* versus Re*(v).*

Plotting $Re\left(u\right)$ versus $Re\left(v\right)$ yields a linear relationship, and using a least-square line fitting, we can determine both g and an estimated value of *α* at 1 kHz as shown in Fig. 8. The magnitude of g, so determined, differs from its estimated value based on the specifications of the current amplifier by about 5%. This is not too surprising considering that the parameter g also includes the gain difference of the lock-in detectors.

From Eq. (7), we have

$Re(u)=-\alpha +\left|g\right|Re(v)$ (11)

**Fig. 9:**
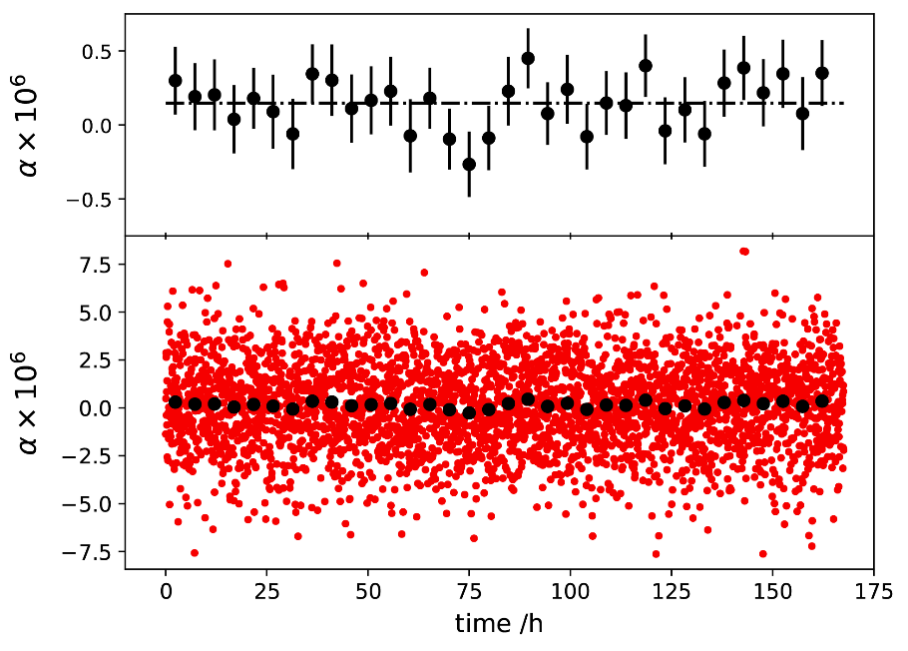
Determined *α* at 1 kHz as a function of time. The black dots were obtained by averaging 100 points, or about 5 hours’ worth of data. The error bars denote the standard errors of the mean of each 100 point segment.

With g known, we can calculate α for each measurement by using$\;\alpha =-Re(u)+\left|g\right|Re(v)$, as shown in Fig. 9. The distribution of the data points is consistent with a constant that is buried in white noise. Each data point takes about 3 min to acquire, and all the data points stay within ±8 × 10^−6^. Averaging 100 points, or about 5 hours’ worth of data, produces a new set of averaged data that fluctuates within ±4 × 10^−7^ about their mean. The fluctuations can be attributed to the limited resolution of the ADC and the timing alignments of the three lock-in detectors.

Similarly, the imaginary component of Eq. (7) can be obtained by

$\beta =-Im\left(u\right)+\left|g\right|Im(v)$ (12)

**Fig. 10:**
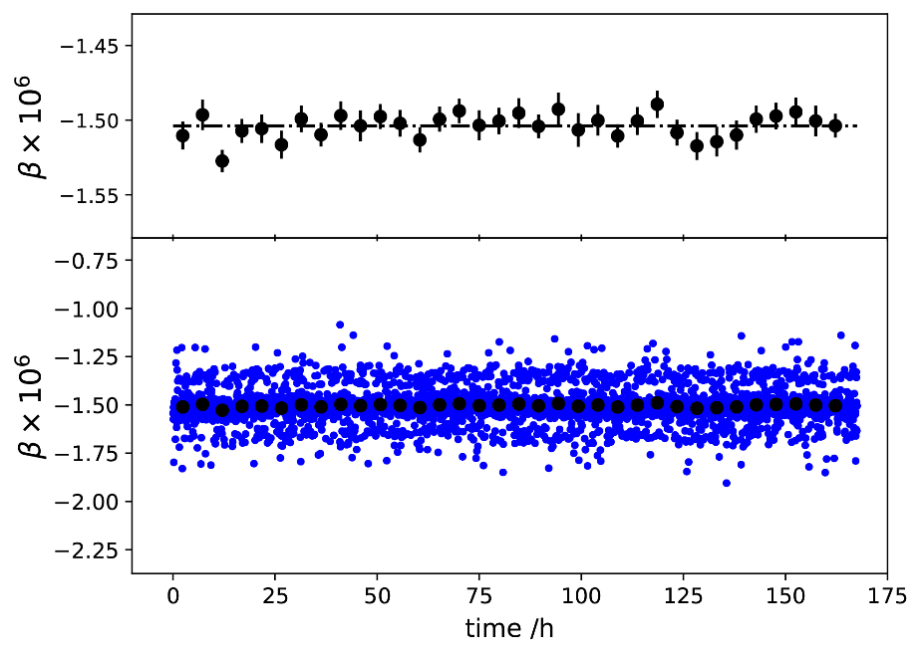
Determined *β* as a function of time. The black dots were obtained by averaging 100 points, or about 5 hours’ worth of data. The error bars denote the standard errors of the mean of each 100 point segment.

Figure 10 shows β as a function of time. Note that the vertical extents of the plots in Fig. 10 are one tenth of the corresponding plots in Fig. 9, clearly demonstrating less noise in the imaginary components, which is a consequence of the fact that the relative phase stability is better than the relative amplitude stability of the two sources.

**Fig. 11:**
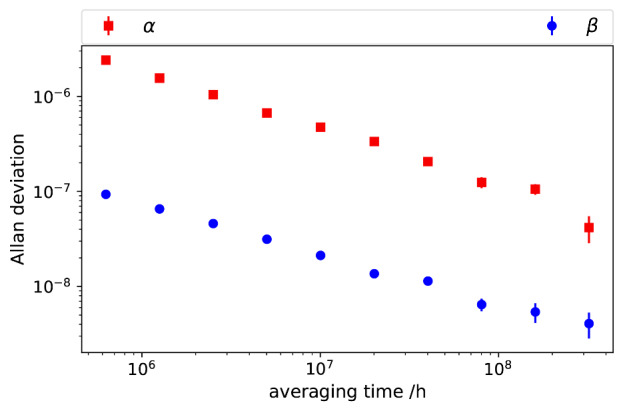
Allan deviations of measured impedance ratio: solid squares for in-phase component and solid circles for quadrature.

The Allan deviations of the measured impedance ratio of *Z_1_* and *Z_2_*, at 1 kHz and an rms value of 0.7071 V, are shown in Fig. 11. The Allan deviations of *α* decrease to below 10^−7^, demonstrating the stability of the digital bridge. The quadrature component shows lower deviations, demonstrating the excellent phase stability of the digital system.

**Fig. 12:**
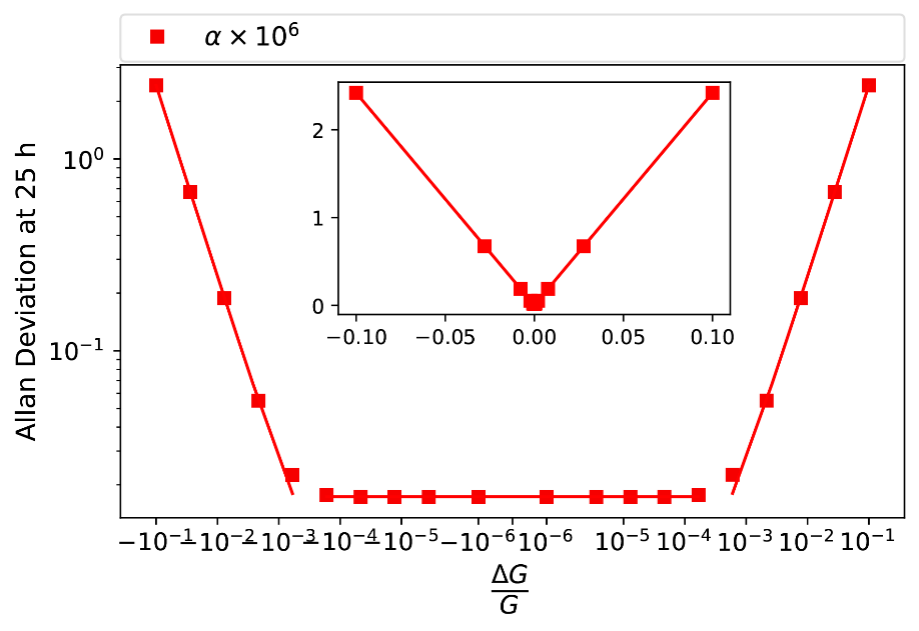
Allan deviation of in-phase resistance ratio as function of *∆G/G*. The inset shows the same data on linear axes.

Effective cancellation of the source noise using Eq. (2) requires precise determination of *G*. The Allan deviation of the in-phase resistance ratio at 25 h is shown in Fig. 12 as a function of fractional magnitude change of *G*, *∆G/G*, at constant phase. The log-log plot shows a broad bottom, indicating that the source noise is largely cancelled out, provided that the magnitude of *G* is determined within a factor of 10^−3^. The Allan deviation increases linearly with the magnitude of large *∆G/G*, as shown in the inserted linear plot.

**Fig. 13:**
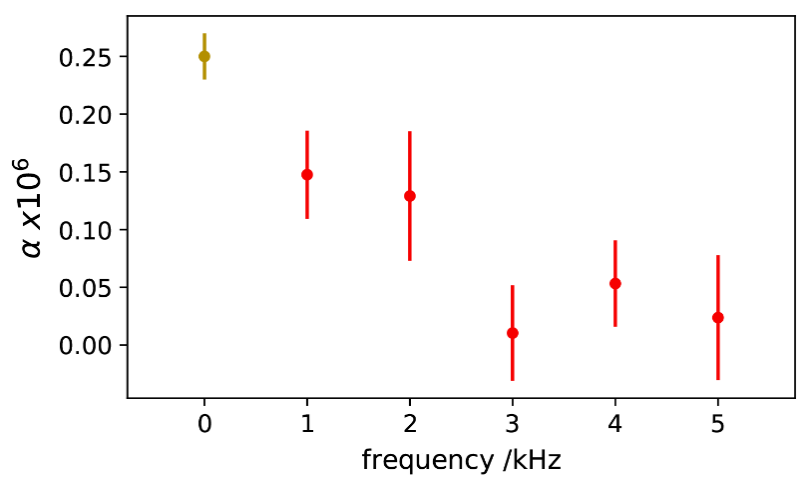
Measured *α* as a function of frequency (red); DC value of *α* (olive) shown for comparison.

Measurements of *α* from 1 kHz to 5 kHz are shown in Fig. 13. Its frequency dependence, based on the AC measurements alone, is buried in the fluctuations of type A noise. However, a comparison with the DC value of *α* is also shown Fig. 13, which was measured with a Measurements International 6010C automatic resistance bridge, and it suggests that the two resistors may have a small difference of frequency dependence. A calibration obtained from the Physikalisch-Technische Bundesanstalt (PTB) for a similar Vishay resistor exhibits a linear frequency dependence of 0.25 × 10^−6^ /kHz.

**Fig. 14:**
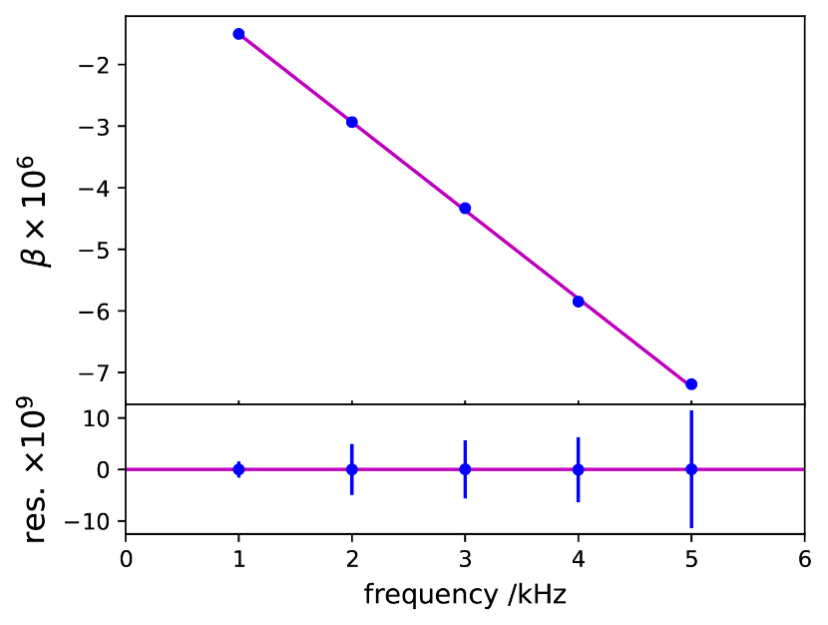
Measured *β* as a function of frequency (top). Residual after fitting with a straight line (bottom).

Measured *β* values as a function of frequency are shown in Fig. 14, exhibiting a linear frequency dependence, which is consistent with a simple parallel RC model for the AC resistors under comparison. The parallel capacitances of the two AC resistors appear to be matched within 0.02 pF. Small residuals that can be seen in the figure after fitting with a straight line demonstrate again the excellent phase stability of the digital bridge.

## 4 Conclusion

We tested a simple digital impedance bridge using two nominally equal resistors to form a 1:1 ratio. In contrast to the conventional approach of emphasizing precision and stability of the voltage sources driving the bridge, we adopted an approach that focused on the resolution and stability of the detectors. Fluctuations of source voltages were largely removed through postprocessing of the digitized data, and the measurement results were limited mainly by the detector noise.

We also experimented with using commercial lock-in amplifiers for AC voltage ratio measurements. Lock-in amplifiers are traditionally used to measure small AC signals, typically deviations from null in AC bridge applications. In this work, we demonstrated a system using three lock-in detectors for measuring small deviations from the perfect AC ratio of unity magnitude, which achieved stabilities and resolutions of 0.1 μV/V within a few hours for each point from 1 kHz to 5 kHz. Compared to the digital sampling bridge using the modified 3458A multimeters, the digital bridge based on the lock-in amplifiers, which require no modification, is easier to use, more robust for remote programming and controlling, and more readily applied over a wide frequency range. However, we also demonstrated the superior stability and resolution at 1 kHz using three 3458A multimeters for measurements of the voltage ratios [7], achieving a signal-to-noise ratio approximately one order of magnitude higher when compared over the same averaging time window with the lock-in system. In the future, we intend to continue experiments with this superior voltage ratio measurement system at other frequencies within the constraints of the equivalent-time sampling principles [9].

The preliminary results reported in the digest paper [7] using the modified 3458A multimeters for measurements of the voltage ratios and the more detailed results presented here using SR860 lock-in detectors establish two important advances towards developing the ultimate detector-limited digital bridge. The 3458A-based bridge proves that at 1 kHz, we can achieve the limiting condition where the overall bridge resolution and stability (0.01 μΩ/Ω in less than 15 min) are limited by the detectors rather than by the excitation sources for the 1:1 comparisons of the two resistors. It should be mentioned that for future RC comparisons, where one of the sources is phase shifted, the effect of the 3458A’s limited bandwidth needs to be carefully investigated because the purity of the source spectra can become critical due to aliasing. The 3458A-based system, however, cannot be easily extended to unique frequencies like 1592 Hz and 1233 Hz due to constraints of the equivalent-time sampling principles. In contrast, the lock-in system works at any frequency in the audio range, but it suffers from a higher level of detection noise, taking a few hours to achieve 0.1 μΩ/Ω type A uncertainty. The detection noise can be attributed to the limited resolution of the ADC and the timing alignments of the three lock-in detectors, which were implemented in the control software using an M_1_-M_2_-M_3_-M_2_-M_1_ sequence for the data readings. This alignment method is valid within timing jitters of the communication bus and has the advantage of being easily implemented with most lock-in detectors. However, significant reduction of the detection noise is expected in the future by implementing a hardware-based timing alignment, which becomes possible with the newest lock-in detectors. Another interesting research topic is to explore modern data-acquisition boards that can be configured and programmed to demodulate at any audio frequency, like SR860, while offering high resolutions and linearities similar to 3458A multimeters.

In order to take advantage of the excellent phase control and stability of the digital bridge and extend the newly demonstrated measurement capability for comparing a capacitor with a resistor, we plan to explore two different approaches. For an impedance ratio with the nominal value of one in magnitude, we plan to adopt the technique proven by Delahaye and Goebel [16], where slight frequency adjustment is allowed, so that the impedance ratio of the capacitor to the resistor is arbitrarily close to one in magnitude with its phase close to 90°. For other impedance ratios, we need to further investigate application of AC voltage scaling and calibration functions in the bridge by adding an inductive voltage divider between one of the high-potential ports and the voltage measurement system, so that the apparent magnitude of the main voltage ratio stays near unity.
